# Variation in global distribution, population structures, and demographic history for four *Trichiurus* cutlassfishes

**DOI:** 10.7717/peerj.12639

**Published:** 2021-12-15

**Authors:** Hsiu-Chin Lin, Chia-Jung Tsai, Hui-Yu Wang

**Affiliations:** 1 Department of Marine Biotechnology and Resources, National Sun Yat-sen University, Kaohsiung, Taiwan; 2 Doctoral Degree Program in Marine Biotechnology, National Sun Yat-Sen University, Kaohsiung, Taiwan; 3 Institute of Oceanography, National Taiwan University, Taipei, Taiwan

**Keywords:** 16S, Bentho-pelagic, Cutlassfish, Fish stock, NW Pacific, Population expansion, Pleistocene

## Abstract

**Background:**

Species-specific information on distribution and demographic patterns provides important implications for conservation and fisheries management. However, such information is often lacking for morphologically-similar species, which may lead to biases in the assessments of these species and even decrease effort towards sustainable management. Here, we aimed to uncover the distribution range, population structure and demographic history for four exploited *Trichiurus* cutlassfishes using genetics. These cutlassfishes contribute substantial global fisheries catch, with a high proportion of catch harvested from the NW Pacific.

**Methods:**

We chose the widely available mitochondrial 16S ribosomal RNA (16S) as the genetic marker for cutlassfishes. We compiled the 16S sequence data from both the GenBank and a survey of trawler catch samples along the NW Pacific coasts 22–39°N. Genealogical relationships within each species was visualized with haplotype networks and potential population differentiations were further evaluated with AMOVA. Demographic histories were estimated using neutrality test, mismatch analysis, and the Bayesian skyline plot. The reconstructed phylogenetic trees were used to delimit and estimate the divergence time of species and included populations.

**Results:**

In each of two cosmopolitan species, *T. lepturus* and *T. nanhaiensis*, we observed distinct populations along the coasts of warm oceans; such population differentiation might result from historical geographic barriers in the Pleistocene. In the NW Pacific, four *Trichiurus* species vary in their distribution habitats, which reflect differential ecological niches among these species. The small-sized* T. brevis* are primarily found in nearshore habitats; the warm-affiliated* T. nanhaiensis* are present along the path of the Kuroshio Current; the cold-affiliated *T. japonicus* spatially diverged from the widely-distributed *T. lepturus*, with the latter mainly occupy in warmer regions. Despite these differences, a single well-mixing fish stock, thus one management unit, was identified in each of the four species, presumably due to expansion of their population sizes predated the Last Glacial Maximum and a lack of distribution barrier. The most dominant *T. japonicus*, which have at least one magnitude higher effective population size than the others, show a unique abrupt size expansion event at 75 to 50-kilo years ago when the low sea level occurred during the ice age.

**Main conclusions:**

The demographic history revealed by our genetic analyses advances understanding of the current distribution and population structure for these congeneric species. Moreover, the uncovered population structure provides insight into the assessment and management of these species. Such information complements contemporary knowledge about these species and enables us to forecast their ability to resist future environmental and anthropogenic disturbances.

## Introduction

Cutlassfish or hairtail (Family Trichiuridae) are broadly distributed in the tropical-to-temperate oceans. They are bentho-pelagic species that present diurnal vertical migration from the seafloor to surface reportedly in synchrony with their prey (Guan et al., 2019; Munekiyo, 1990). They contribute substantial global fisheries catch (>1.2 million tons per year, FAO FishStat, 2016, available at: http://www.fao.org/fishery/species/2468/en) and are especially popular for coastal fisheries of the NW Pacific countries, including China, Japan, Korea, and Taiwan. Similar to most commercially important species, the global and regional catch of cutlassfishes are both reducing drastically likely due to long-term fishing (Chiou et al., 2006; Memon et al., 2016). In the NW Pacific, at least ten cutlassfish species have been recorded, including *Benthodesmus tenuis*, *Eupleurogrammus muticus*, *Evoxymetopon poeyi*, *Evoxymetopon taenietus*, *Lepturacanthus savala*, *Tentoriceps cristatus*, *Trichiurus brevis*, *T. japonicus*, *T. lepturus*, and *T. nanhaiensis* (FishBase). Among these species, *T. japonicus* is the dominant and most exploited, followed by *T. nanhaiensis* (Wang, Dong & Lin, 2017). The NW Pacific *T. japonicus* was considered as a subspecies of the cosmopolitan species *T. lepturus*, but distinctness between these two species has been corroborated by both morphometric and genetic data (Chakraborty, Aranishi & Iwatsuki, 2006b; Tzeng, Chen & Chiu, 2007). Besides *T. japonicus, T. lepturus*, and *T. nanhaiensis*, the remaining species have little to no commercial value because their flesh is scanty.

Spatiotemporal information on species distribution and demographic structure is required for fisheries management. However, such information is often lacking for those morphologically-similar species. Molecular markers have been widely used to identify genetic variation and structure among individuals for fishery species (Abdul-Muneer, 2014). The detection of genetic differentiation suggests that the study species comprise different populations (Carvalho & Hauser, 1995), which should be regarded as separate management units (Moritz, 1994). The very first population genetic study of NW Pacific cutlassfishes was conducted with 11 allozyme markers (Wang et al., 1994). Although the authors reported population differentiation within each of these examined species: *T. brevis*, *T. japonicus*, and *T. nanhaiensis*, their computation of genetic distances (D) among populations were incomparable to the later developed metric such as G_ST_ (Culley et al., 2002); thus, the reported intra-specific population structure for these species by Wang et al. (1994) remains to be confirmed. In addition, it is challenging to properly compare the allozyme heterozygosity values and differentiation pattern across taxa partially because of the inconsistency among types of enzymes (Ward, Skibinski & Woodwark, 1992). In recent years, Polymerase Chain Reaction (PCR) technique-based genetic markers, which comprise a higher amount of polymorphism that enable to estimate demographic history, are much more widely applied. Such markers include mitochondrial DNA and nuclear microsatellite DNA markers (Allendorf, 2017; Avise, 2009; Schlötterer, 2004).

In *T. japonicus*, magnitudes of genetic diversity and population differentiation are equivocal among studies using different genetic markers. Based on either the mitochondrial control region (Xiao et al., 2014) or cytochrome b (cyt b) DNA sequences (He et al., 2014), a single population was observed between the East and South China Seas along the coast of China with potential expansion of population size in the late Pleistocene (70–216 kilo years ago, kya). However, also based on cyt b yet with broader coverage of sampling sites, a later study observed two genetically differentiated populations between the East and South China Seas, with Taiwan Strait as the transient zone (Tzeng et al., 2016). This population structure pattern is mostly supported by allozyme markers (Wang et al., 1994), except an additional northern population in the Yellow Sea is observed based on allozyme. The allozyme genetic distances between the population in the Yellow Sea and the others ranged from 0.017 to 0.048 (Wang et al., 1994).

Except for the dominant species *T. japonicus*, present knowledge of population genetics and demographic history for other common NW Pacific cutlassfish species is still limited. Warm-affiliated species *Trichiurus nanhaiensis* has wide geographical distribution in the Indo-Pacific Ocean with the northernmost reported location at Miyazaki, Japan (Chakraborty, Aranishi & Iwatsuki, 2006a). However, only limited data on its population structure along the coast of southern China with mitochondrial cyt b and 11 allozyme markers are available (He et al., 2014; Wang et al., 1994). Both types of markers identified two populations, but only the allozyme markers revealed differentiation corresponding to geographic locations (genetic distance of 0.015 between Sanya, Hainan and Santo, Guangdong). A circa 100-kilo years of gradual declines of effective population size, followed by a rapid expansion during 15–37.5 kya were estimated based on cyt b (He et al., 2014). For another warm-affiliated species *T. brevis*, population genetic data based on allozyme markers are limited from a few adjacent locations in the coasts of China of the South China Sea (Wang et al., 1994). A recent study applied both mitochondrial COI gene and 13 microsatellite loci as the markers to infer the intra-specific genetic variation of *L. savala* and found no clear pattern in relation to geographic areas along the coast of China (Gu et al., 2021).

To advance the current understanding of the population genetics for cutlassfishes, we applied a widely used cutlassfish genetic marker, partial DNA sequence of mitochondrial 16S ribosomal RNA (rRNA), to explore the intra-specific genetic variation and reconstruct the demographic history of four *Trichiurus* species: *T. brevis*, *T. japonicus*, *T. lepturus*, and *T. nanhaiensis*. We took advantage of 16S rRNA marker which has a worldwide sampling coverage of the two cosmopolitan species *T. lepturus* and *T. nanhaiensis* on GenBank by combining with our catch samples into the analyses. Our comparative study of these congeneric species will mark their inter-specific variation in distribution ranges, population structure, and demographic history across global oceans, providing invaluable information for sustainable management of cutlassfish fisheries.

## Materials & Methods

### Sampling and DNA data

**Figure 1:**
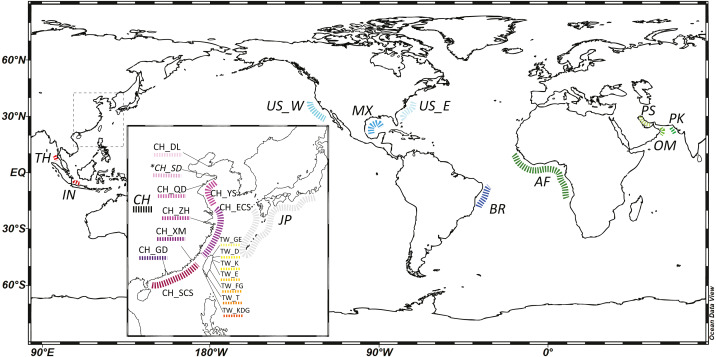
A map of localities of fishery (normal font) and GenBank samples (italic font) in this study. An inset map shows detailed localities of samples in the Northwest Pacific region. Abbreviation of localities and detailed information are listed in Table 1 and Table S1. *: Both fishery and GenBank samples. The map was made with Ocean Data View (https://odv.awi.de).

To comprehend species distribution and intra-specific population genetic structure, we combined available fish samples and genetic data for the cutlassfishes from two sources: (1) cutlassfish samples from trawler fisheries catch in various landing ports in China and Taiwan along the coast of NW Pacific following the sampling protocols in Wang, Dong & Lin (2017), (2) GenBank DNA sequences of cutlassfishes from Japan, China, Thailand, and Indonesia in the West Pacific; USA in the East Pacific; USA and Brazil in the West Atlantic; Angola, Benin, Bissau, Gabon, and Guinea in the East Atlantic; Iran, Oman, and Pakistan in the Indian Ocean (see Table S1). Together, these data provide global coverage for four NW Pacific common cutlassfishes: *Trichiurus brevis*, *T. japonicus*, *T. lepturus*, and *T. nanhaiensis* (Table 1, Fig. 1).

**Table 1:** Sampling information for the four cutlassfish species analyzed in this study. Please see Table S1 for the detailed information of samples downloaded from GenBank.

	Latitude	Longitude	*T. brevis*	*T. japonicus*	*T. lepturus*	*T. nanhaiensis*
Fishery samples						
CH_DL	38.912	121.602	0	9	0	0
CH_SD	36.891	122.449	0	16	0	0
CH_QD	36.066	120.369	45	5	0	0
CH_ZH	29.989	122.205	2	144	0	0
CH_XM	24.330	118.196	42	0	0	0
CH_GD	22.716	115.767	12	0	0	0
TW_GE	25.130	121.767	0	29	0	2
TW_D	24.970	121.980	0	49	1	0
TW_K	24.907	121.862	0	51	1	0
TW_E	24.501	121.850	0	58	4	23
TW_FG	22.791	121.192	0	11	13	0
TW_T	22.725	120.252	0	294	46	20
TW_KDG	22.850	120.087	0	150	37	52
GenBank samples						
West Pacific						
JP	–	–	0	11	0	10
CH_YS	–	–	0	6	0	0
CH_ECS	–	–	0	16	0	0
CH_SCS	–	–	1	4	11	15
CH	–	–	0	16	0	0
TH	–	–	0	0	0	2
IN	–	–	0	0	0	6
East Pacific						
US_W	–	–	0	0	1	0
West Atlantic						
US_E	–	–	0	0	5	0
MX	–	–	0	0	2	0
BR	–	–	0	0	10	0
East Atlantic						
AF	–	–	0	0	10	0
Indian Ocean						
PS	–	–	0	0	0	7
OM	–	–	0	0	3	8
PK	–	–	0	0	0	5

Total genomic DNA was extracted from soft tissue of each catch sample using BioKit (Miaoli, Taiwan) Tissue & Cell Genomic DNA Purification Kit following the manufacturer’s instructions. Partial sequences of mitochondrial gene 16S ribosomal RNA (16S) were amplified by polymerase chain reaction (PCR) as previously described (Miya & Nishida, 1996). Direct sequencing of the purified PCR products was performed on an ABI 3730XL Genetic Analyzer with BigDye terminator cycle sequencing reagents (Applied Biosystems, Foster City, CA, USA).

### Intra-specific genetic variation

16S data matrix of each species was individually aligned with CLUSTAL X (Thompson et al., 1997) using default settings and adjusted manually in Geneious 8.1.8 (https://www.geneious.com). The best nucleotide substitution models were Jukes-Cantor (JC) model (Jukes & Cantor, 1969) for *T. lepturus*; Kimura two-Parameter (K2P) model (Kimura, 1980) for *T. brevis*, *T. japonicus*, and *T. nanhaiensis* selected in MEGAX based on the Bayesian Information Criterion (BIC). Gaps were treated as missing data and the matrix was trimmed to the longest shared length. Haplotype network analyses were conducted in PopART (Population Analysis with Reticulate Trees) (Leigh & Bryant, 2015) with TCS (Clement, Posada & Crandall, 2000) as the network inference method. When differentiation was observed within species, Analysis of Molecular Variance (AMOVA) was conducted to estimate the *F*-statistics and test for differentiation among groups of populations. The best substitution model was applied and the significance of these values was estimated with 1,000 permutations as implemented in Arlequin (Excoffier & Schneider, 2005). Standard genetic diversity indices, including number of haplotypes (Nh), haplotype diversity (*h*), and nucleotide diversity (*π*) were estimated for each species and population using DnaSP v6 (Excoffier & Schneider, 2005).

### Demographic history

Neutrality statistics, the sequence mismatch analysis (MMA), and Bayesian skyline plots (BSP) were applied to estimate the demographic history of the study species or groups with samples size higher than 100. Neutrality tests including Tajima’s *D* (Tajima, 1989) and Fu’s *F*_*S*_ (Fu, 1997) were computed for each species and included groups using DnaSP. Significance levels of these statistics were set and interpreted following the manual (Fay & Wu, 1999; Hein, Schierup & Wiuf, 2004; Nielsen, 2001; Simonsen, Churchill & Aquadro, 1995; Tajima, 1989).

Signals of sudden population size change were detected using MMA implemented in Arlequin (Rogers & Harpending, 1992; Schneider & Excoffier, 1999). The raggedness index that quantified the smoothness of the observed mismatch distribution (*i.e.,* differences between pairs of sequences) and the sum of square deviations (SSD) between the observed and expected mismatch under a size expansion model was also calculated (Harpending, 1994; Harpending et al., 1993). Three parameters were estimated with coalescence simulations under an expansion model, *τ* (the time since expansion), *θ*_0_ (population size before an expansion), and *θ*_1_(population size after an expansion).

The coalescent BSP (Drummond et al., 2005) method was conducted to estimate change of effective population size through time using BEAST v1.10.4 (Barido-Sottani et al., 2017; Drummond & Rambaut, 2007; Suchard et al., 2018). The configuration files were first generated with BEAUti v1.10.4 (Drummond & Rambaut, 2007) and run for 50 million generations (except 500 million generations for *T. japonicus* due to the large sample size) under the best nucleotide substitution model for each species, respectively. A strict clock model was selected (Drummond et al., 2006; Ho & Shapiro, 2011) and the timings obtained here need to be taken as an approximation because a well-calibrated molecular clock for 16S in cutlassfish is not available; thus, a clock rate of electric fish was applied instead (Alves-Gomes, 1999). A coalescent Bayesian skyline tree model with 10 groups and uniform priors was applied. The chain was sampled every 1,000 generations (except 10,000 generations for *T. japonicus* for reasonable computation time) and the first 5 million generations (100 million generations for *T. japonicus*) were discarded as burn-in. Trace plots were inspected in Tracer v1.7.1 (Rambaut et al., 2018) and the results of the three replicate runs were combined with LogCombiner v1.10.4 (Drummond & Rambaut, 2007). The past population dynamics were then analyzed and performed in Tracer.

### Phylogenetic relationships and divergence time estimation

Because broader taxon coverage can improve the accuracy of phylogenetic inference, eight con-familial species with available 16S sequences from GenBank were included for the following phylogenetic analyses (Table S1). Sequences of three non-trichiurid scombroids *Scombrolabrax heterolepis* (Scombrolabracidae), *Thyrsitoides marleyi* (Gempylidae), and *Prometichthys prometheus* (Gempylidae) were also included and assigned as the outgroup based on the phylogenomic framework of the pelagic fish families (Friedman et al., 2019; Miya et al., 2013). To avoid the redundancy of identical sequences, only haplotypes for each of the four study species were included in the analyses. The combined data matrix was then aligned with CLUSTAL X (Thompson et al., 1997) using default settings and adjusted manually in Geneious 8.1.8 (https://www.geneious.com). Complete deletion of aligned sites with gap(s) was set for the following analyses because rRNA sequences presented some alignment difficulties due to the presence of insertions and deletions. The nucleotide substitution model GTR+I+G (Nei & Kumar, 2000) was selected with the program MEGA on the basis of the Bayesian Information Criterion (BIC). A discrete Gamma distribution was used to model evolutionary rate differences among sites (5 categories (+G, parameter = 0.4497)). The rate variation model allowed some sites to be evolutionarily invariable ([+I], 29.78% sites). Maximum Likelihood (ML) and Bayesian Inference (BI) methods were applied to reconstruct the phylogenetic relationships under the CIPRES Science Portal v2.2 (Miller, Pfeiffer & Schwartz, 2010). The ML tree search was conducted with Nearest-Neighbor-Interchange (NNI) starting with a tree generated by the Neighbor-Joining method in IQ-TREE (Trifinopoulos et al., 2016). The best substitution model was applied and the node support values were estimated with 1,000 bootstrapped replicates. The BI tree search was run for 10 million generations and sampled every 1,000 generations in MrBayes v3.1.2 (Huelsenbeck & Ronquist, 2001). Two simulated independent runs each comprised of four chains were conducted. The sampled parameter values were evaluated in Tracer and the first 20% generations were discarded as burn-in. Trees of the two runs after burn-in were pooled by LogCombiner, and the posterior probability of each node and the mean branch lengths on maximum clade credibility (MCC) tree were calculated with TreeAnnotator v1.10.4 (Drummond & Rambaut, 2007). In addition, we employed distance-based Automated Barcoding Gap Discovery (ABGD; Puillandre et al., 2012) to delimit the four *Trichiurus* species. ABGD was conducted with the Kimura model (K80) (Kimura, 1980) and default settings for the remaining parameters.

Divergence time was estimated in BEAST with the configuration files generated with BEAUti assuming a strict molecular clock model and Yule-process-speciation prior to the branching rate (Drummond et al., 2006). As in the phylogenetic analyses, the best substitution model was applied. The MCC tree retrieved earlier was assigned as the starting tree. The node of the Most Recent Common Ancestor (MRCA) of all trichiurids was set to a normal prior distribution with either mean of 62 mya, stdev of 0.5, or mean of 45 mya, stdev of 2 based on two taxon-wide time trees reconstructed with comprehensive sequence datasets of mitogenome (min 61.97, max 63.20; Miya et al., 2013) and genome (MCMCtree kmeans min 41.65, max 49.88; Friedman et al., 2019), respectively. Two simulated independent analyses each run for 100 million generations, sampled every 1,000 generations were conducted. The sampled parameter values were evaluated in Tracer, and the first 20% generations were discarded as burn-in. Trees of the two runs after burn-in were pooled by LogCombiner, re-sampled every 10,000 generations, and the posterior probability density was summarized on an MCC tree with TreeAnnotator.

## Results

### DNA data reveal distinct distributions ranges among cutlassfishes

101 *Trichiurus brevis*, 820 *T. japonicus*, 114 *T. lepturus*, and 83 *T. nanhaiensis* individuals were collected throughout the NW Pacific (Table 1, Fig. 1). 16s rRNA partial sequences of about 530 based pairs (bp) were retrieved. Combining and aligning the con-specific sequences from GenBank (Table S1), a total of 13, 65, 14, 27 haplotypes were identified in 102 *T. brevis*, 872 *T. japonicus*, 144 *T. lepturus*, and 150 *T. nanhaiensis* individuals (Table 2 and Table S1). *T. lepturus* were primarily recorded from the warm oceans, including tropical and temperate coasts of the Atlantic, Pacific, and Indian Oceans. *T. nanhaiensis* were also mainly recorded from the warm regions of the Indo-Pacific Oceans, with the northernmost recorded site in Miyazaki at Kyushu Island, Japan, but were absent in the East China Sea along the coast of China (Table S1). Compared to those two cosmopolitan species, *T. japonicus* were primarily distributed in the NW Pacific; northernmost and southernmost records are located in Dalian, China and the northern South China Sea, respectively (Table S1). Available samples indicated *T. brevis* co-occur with *T. japonicus* along the coast of China but are absent in Taiwan.

**Table 2:** Sample size (n), genetic diversity including number of haplotypes (Nh), haplotype diversity (h), nucleotide diversity (*π*), neutrality tests including Tajima’s D and Fu’s Fs, mismatch distribution test including *τ*, *θ*_0_, *θ*_1_, Harpending’s raggedness index, and sum of squared differences (SSD) for each species and intra-specific group.

			Diversity				Neutrality test			Mismatch distribution test				
Species/groups	n		Nh	h	*π*		D	Fs		*τ*	*θ*_0_	*θ*_1_	Raggedness index	SSD
*T. brevis*	102		13	0.2895	0.00069		−2.3128^***^	−17.5143^***^		3.000	0.000	0.417	0.26303	0.00098
*T. japonicus*	872		65	0.4722	0.00147		−2.4872^***^	−137.425^***^		0.643	0.000	99999	0.10058	0.00026
*T. lepturus*	144		14	0.4199	0.00447		−0.8180	−1.1404		3.000	0.000	0.512	0.30946	0.07916
Group 1	116		5	0.1299	0.00031		−1.6261^**^	−2.1388		3.000	0.000	0.109	0.67736	0.00328
Group 2	10		5	0.7556	0.00345									
*T. nanhaiensis*	150		27	0.7121	0.00524		−1.6329^*^	−16.5324^***^		9.472	0.000	2.367	0.03480	0.01022
Group 1	122		24	0.7194	0.00514		−1.6293^*^	−13.7893^***^		0.814	0.000	99999	0.07808	0.00162
Group 2	28		13	0.9153	0.00786									

**Notes.**

* *P* < 0.05 (0.02 in Fs).

** *P* < 0.01.

*** *P* < 0.001.

### Distinct intra-specific population structures among cutlassfishes

**Figure 2:**
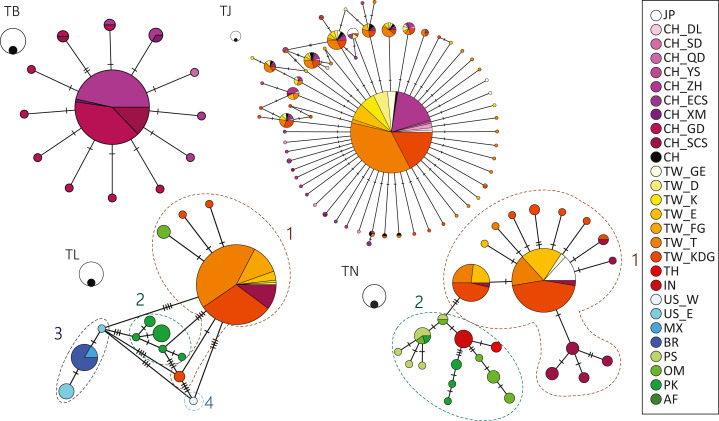
Haplotype network showing the haplotype connections and frequency of geographic occurrence in the four cutlassfish species based on 16S. Circles represent different haplotypes; sizes of circles correspond to observed frequencies of haplotypes, with the black circles scale to 1 and white circles scale to 10 samples. Each bar represents one mutational change between the connected haplotypes. Colors correspond to different sampling localities shown in the legend on the right. Numbers by dashed circles are groups in species. TB: *T. brevis*, TJ: *T. japonicus*, TL: *T. lepturus*, TN: *T. nanhaiensis*.

No differentiation was observed in the haplotype network results for both *T. japonicus* and *T. brevis* in the NW Pacific (Fig. 2). A major and potential ancestral haplotype was shared by 631 (72%) individuals from the East China Sea and Taiwan for *T. japonicus* (Table S1). Similarly, a major and potential ancestral haplotype was shared by 76 (75%) individuals from the East China Sea and the northern South China Sea (Table S1) for *T. brevis*.

On the contrary, differentiation was observed in *T. lepturus* and *T. nanhaiensis* among warm ocean regions. In *T. lepturus*, differentiation of four populations was observed in the haplotype network result, corresponding to Indo-West Pacific (Group 1), Southeast Atlantic (Group 2), West Atlantic (Group 3), and Northeast Pacific (Group 4) (Fig. 2, Table S1). AMOVA results showed 95.17% of the variance was among the four groups and F_ST_ value of 0.95 was highly significant (*P* < 0.001). For the haplotypes in Group 1, a major one was shared by 144 (76%) individuals throughout Taiwan and the South China Sea (Table S1). For the haplotypes in Group 3, a major one was shared by individuals from the coast of Mexico and Brazil (Table S1). In *T. nanhaiensis*, differentiation of two populations was observed in the haplotype network result, corresponding to West Pacific (Group 1) and Indian Ocean (Group 2) (Fig. 2, Table S1). AMOVA results showed 69.42% of the variance was among the two groups and F_ST_ value of 0.69 was highly significant (*P* < 0.001). Two major haplotypes were observed in Group 1 and both were shared by individuals from Japan, Taiwan, and the South China Sea. These species varied in both haplotype and nucleotide diversity: *T. nanhaiensis* > *T. japonicus*, *T. lepturus* > *T. brevis* (Table 2). Note that differential genetic diversity occurred between 2 groups in each of *T. lepturus* and *T. nanhaiensis*.

### Demographic history

For neutrality tests, most species and groups had significantly negative Tajima’s D and Fu’s Fs values, which suggest a recent population expansion, except *T. lepturus* (Table 2), which their observed mismatch distribution showed multiple peaks (Fig. S1). For all species and groups, their Raggedness indices and SSD values were all insignificantly different from the expansion model (Table 2). The size differences before (*θ*_0_) and after (*θ*_1_) the expansion event were especially huge (*θ*_0_ =0, *θ*_1_ =99999) in *T. japonicus* and *T. nanhaiensis* Group 1, negligible in *T. brevis*, *T. lepturus* and *T. lepturus* Group 1, and in between for the others (Table 2). The expansion events happened earlier in *T. nanhaiensis* (*τ* = 9.472), and much more recent in *T. japonicus* and *T. nanhaiensis* Group 1 (*τ* = 0.643 and 0.814) (Table 2).

**Figure 3:**
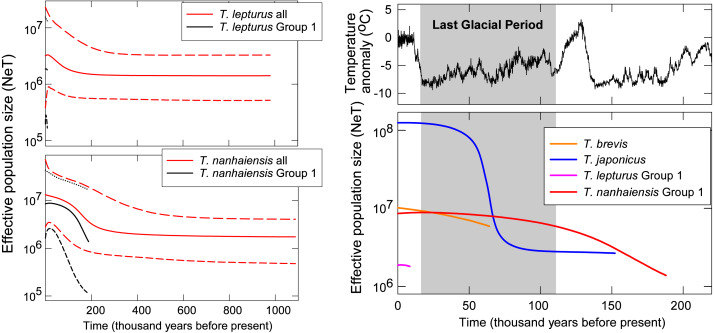
Coalescent Bayesian skyline plots of *Trichiurus lepturus* (upper left), *T. nanhaiensis* (lower left), and the NW Pacific population for the four study species (right). The lines represent the mediums (solid lines), and the upper and lower bounds (dash lines) of the 95% highest posterior density (HPD) interval. Historic temperature anomaly to present time was estimated from the Vostok Ice Core (Jouzel et al., 1993; Jouzel et al., 1987; Jouzel et al., 1996; Petit et al., 1999).

Gradual population growth since the time of the most recent common ancestor was shown in the coalescent BSP for *T. brevis*, *T. lepturus*, *T. nanhaiensis*, *T. nanhaiensis* Group 1, but relatively stable for *T. lepturus* Group 1 (Fig. 3). A sudden population expansion of effective population size from around 5 × 10^6^ to 10^8^ was observed in *T. japonicus* around 70 to 50-kilo years before present (Fig. 3). The current effective population sizes of the two cosmopolitan species *T. lepturus* and *T. nanhaiensis* were 3.2 × 10^6^ and 1.3 × 10^7^, respectively (Fig. 3). In the NW Pacific, effective population sizes were largest in *T. japonicus* (1.2 × 10^8^), intermediate in *T. brevis* (10^7^) and Group 1 of *T. nanhaiensis* (8.6 × 10^6^), and smallest in Group 1 of *T. lepturus*, 1.8 ×10^6^ (Fig. 3).

**Figure 4:**
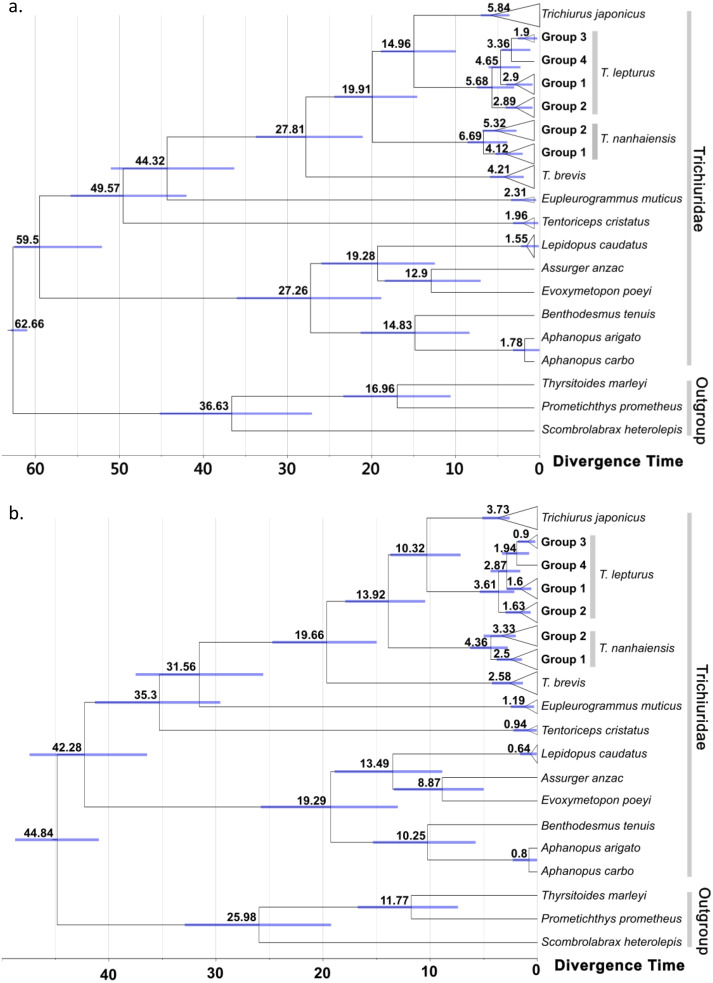
Time-calibrated phylogeny summarized on the maximum clad credibility (MCC) tree from Bayesian analyses. Calibration constrain was set to the most recent common ancestor of trichiurid species following (A) Miya et al. (2013) and (B) Friedman et al. (2019), respectively. The number and bar around each node represent the estimated age and its 95% highest posterior density (HPD) in million years. Group labels followed the haplotype network division in Fig. 2.

### Phylogeny and time tree

No conflicts were observed for well-supported clades in the ML (node support > 75) and BI trees (>85) (Figs. S2 and S3). The score of the best ML tree was −3025.638. In both trees, the twelve trichiurid species and the genus *Trichiurus* were respectively grouped as a well-supported monophyletic group when multiple haplotypes were included. The trichiurid genus-level relationships were partially resolved. In *Trichiurus*, *T. brevis* was the basal clade, followed by *T. nanhaiensis*, and *T. japonicus* and *T. lepturus* were sister clades. In *T. lepturus*, two (Groups 2 and 3) of the three groups displayed multiple haplotypes each formed a monophyletic group with high supporting values (ML: Group 2 90%, Group 3 98%; BI: Group 2 0.99, Group 3 0.99). Groups 3 and 4 were sister taxa with high supporting values (ML: 98%; BI: 0.97), and these groups together formed a sister clade with Group 2 in ML (77%). In *T. nanhaiensis*, Group 1 formed a highly-supported monophyletic group (ML: 77%; BI: 0.96) nested inside Group 2. The results of ABGD further confirmed the delimitation of the four *Trichiurus* species, with the inter-specific distance (0.042–0.106) has a barcode gap with the intra-specific distance (0.00–0.024) (Fig. S4). Based on the constrained age prior of Miya et al. (2013) (Fig. 4A) and Friedman et al. (2019) (Fig. 4B), the divergence time for trichurids were estimated and the median age of MRCA of each the four study species ranged from 6.69 to 4.21 and 4.36 to 2.58 mya, respectively (Fig. 4 and Table S2).

## Discussion

### Comparison of the four *Trichiurus* cutlassfishes

Our study has the most comprehensive sampling sites of the four study cutlassfishes to date by integrating global con-specific 16S rRNA sequences from the GenBank and a large number of catch samples from the NW Pacific. Such a large quantity of genetic data provide a high-resolution analysis of inter-specific variation; *e.g.*, our analysis reveals differential distribution habitats among the four *Trichiurus* species in the NW Pacific. The distribution range of *T. japonicus* includes the furthest north at Dalian, China, suggesting capability of tolerating cold for this species. *T. brevis* co-occur with *T. japonicus* along the coast of China but were not found in the adjacent Taiwan even at the same latitude. On the contrary, *T. lepturus* and *T. nanhaiensis* are only present in Taiwan, but not along the China coast. Such differential distribution patterns are compatible with existing knowledge of life histories for these species and the oceanic currents in the NW Pacific. We suspect that the small-sized *T. brevis* (Burhanuddin et al., 2002) and presumably being less capable of swimming a long distance and less aggression compared to *T. lepturus* and *T. nanhaiensis*, tend to stay close to shore where predators are less abundant (Munsch, Cordell & Toft, 2016). In addition, the distribution range of *T. nanhaiensis* covers the southern coast of Kyusyu, Japan, but not along the adjacent China coast in the East China Sea; this indicates the influence of the Kuroshio Current on this warm-affiliated species. Furthermore, *T. lepturus* and *T. japonicus* are sister species with resembled appearance, but they co-occur only in the warm region along the coast of Taiwan and in the South China Sea.

Although the cosmopolitan *T. lepturus* and *T. nanhaiensis* showed intra-specific differentiation across warm ocean regions, neither of these species displayed distinct populations in the NW Pacific. This is also shown in the other two NW Pacific species, *T. brevis* and *T. japonicus*. Together, these results suggest a genetically well-mixture and no barrier for these continental shelf dwellers. Consequently, the NW Pacific cutlassfishes should be treated as a single unit for fisheries management and assessment.

Our results reveal moderately high genetic diversity and large effective population sizes in the three exploited species: *T. japonicus*, *T. lepturus*, and *T. nanhaiensis*. For example, the species-level indices of haplotype and nucleotide diversities for these cutlassfishes generally are close or higher than the median (*h* = 0.70130, *π* = 0.00356) of animals in another mitochondrial DNA marker cytochrome C oxidase I (Goodall-Copestake, Tarling & Murphy, 2012). The estimated total effective population sizes in the NW Pacific are the highest in *T. japonicus* (1.2 × 10^8^), intermediate in *T. nanhaiensis* (8.6 × 10^6^), and the lowest in *T. lepturus* (1.8 × 10^6^); such variation is consistent with the relative abundance in the fisheries catch for these species (Wang, Dong & Lin, 2017). The observed moderate genetic diversity and large population sizes reveal their general resistance to long-term fisheries exploitation in the NW Pacific area (Szuwalski et al., 2017).

In the NW Pacific, a consistent population growth/expansion was observed for all four species based on some if not all the results of the neutrality test, MMA, and BSP. However, the expansion timings vary among species and may not correspond to the warmer, suitable climate after the glacial maxima (LGM, around 20 kya; Ni et al., 2014). The size expansion of these *Trichiurus* species predated the LGM (Fig. 3); thus, we did not observe influences of the last glacial period with fluctuation of sea level or primary productivity on size as suggested by He et al. (2014). Pre-LGM expansion is also observed in another con-familial cutlassfish species *Lepturacanthus savala* along the coast of China (Gu et al., 2021) and a bentho-pelagic fishery species, silver pomfret *Pampus argenteus* from the Indo-Pacific region (Ni et al., 2014; Zhao et al., 2011). Causes of the pre-LGM population expansion for these fishes could be an interesting subject for future studies.

Of note is that marker choice and mutation rate setting can compromise the accuracies of historic population size estimation based on the methods used in this study (Karl et al., 2012). 16S rRNA and cyt b are both widely used in cutlassfish population genetic studies and viewed as useful mitochondrial DNA markers in assessing fish stocks (Baharum, 2012). Yet the former marker applied in this study showed some constraints in revealing minor genetic structures in cutlassfish probably because of the lower variability (Nicolas et al., 2012).

### Global species *Trichiurus lepturus*

For the cosmopolitan species *T. lepturus*, we identified four groups across global oceans: one in the Indo-West Pacific (Group 1), one in the Southeast Atlantic (Group 2), one in the West Atlantic (Group 3), and one in the Northeast Pacific (Group 4), which might be differentiated by historic geographic barriers in the Pleistocene. The closure of Panama Isthmus at 3.5–3 mya (Coates et al., 1992) could be a geographic barrier that separated Group 3 in the West Atlantic from Group 4 in the East Pacific around 3.36–1.94 mya. Chakraborty & Iwatsuki (2006) identified three groups geographically correspond to the Group 1–3 in this study. However, we did not observe the sister relationships of Group 2 and 3 as suggested by Chakraborty & Iwatsuki (2006); disparity between these studies could be due to the lack of sampling for Group 1 and 4 in their study.

Incongruent demographic histories were proposed by the results of the neutrality test, MMA, and BSP for *T. lepturus* and the included Groups 1 and 2. Although both MMA and BSP are widely applied to depict the population size change through time, inconsistency is not uncommon (Grant, 2015). MMA is only based on the nucleotide differences between pairs of individuals, therefore is suggested to be less accurate compared to the signal-enriched genealogy-based method BSP (Felsenstein, 1992; Grant, 2015). The power of the Raggedness Index in MMA has also been questioned (Ramos-Onsins & Rozas, 2002). Therefore, even *T. lepturus* and the included Group 1 are insignificantly different from the population expansion model in MMA, we suspected stable size through time based on the insignificant departure from neutrality and flat BSPs for Group 1. For the total *T. lepturus*, the population size expands about twice starting from around 20,000 years ago based on BSP. However, this might be misled by the pooling of the four populations with genetic heterogeneity (Grant, 2015). The evolutionary relationships among the four groups and group-specific demographic history shall be greatly elucidated by incorporating more samples throughout the full distribution range and multi-loci markers, especially for Groups 3 and 4 along the coasts of America.

### Indo-Pacific species *Trichiurus nanhaiensis*

*Trichiurus nanhaiensis* has a long history of misidentification as *T. lepturus* due to their similar appearance except for the yellowish-green dorsal fin color when fresh for the former (Hsu et al., 2007; Hsu et al., 2009; Shih, Hsu & Ni, 2011). Therefore, DNA-barcoding method applied in this study enables unambiguous species assignment (Kuparinen & Hutchings, 2019; Tzeng & Chiu, 2012; Wang, Dong & Lin, 2017). We reported the distribution range and historic demography of the two populationsin in *T. nanhaiensis*: one population locates in the West Pacific (Group 1), and the other one locates in the Indian Ocean (Group 2). Although only the monophyly of Group 1 is supported using tree methods, division between these two *T. nanhaiensis* populations is significantly supported by the AMOVA and F_ST_ results. Their divergence corresponds to a well-recognized historical Indo-Pacific Barrier (IPB) constituted by the exposed land of the Sunda Shelf and the Sahul shelf during periods of low sea level at Pleistocene, a confirmed separation mechanism for continental shelf species in the Pacific and Indian Oceans (Fleminger, 1986; Rocha, Craig & Bowen, 2007). Group 2 likely has an older MRCA than Group 1 because of the earlier divergence in trees and higher genetic diversity, even it has much fewer samples. This suggests a potential eastward dispersal route from the Indian (Group 2) to the West Pacific Ocean (Group 1). A significant size expansion event in Group 1 which happened since 190 kya can help colonize the large West Pacific area. However, we did not observe further differentiation in the northern South China Sea as suggested by another mitochondrial marker cyt b and allozyme data, though the differentiation pattern of these two markers are not congruent with each other (He et al., 2014; Wang et al., 1994).

### NW Pacific-dominant *Trichiurus japonicus*

*Trichiurus japonicus* is the dominant species in the NW Pacific, with the estimated present effective population size of 1.2 × 10^8^ at least one magnitude higher than the other co-occurring species. This value is about one magnitude higher than the one (3 × 10^7^) estimated based on another mitochondrial marker cytochrome b (He et al., 2014); disparity between these studies might be related to the differences in marker choice and mutation rate setting (Karl et al., 2012). Undoubtedly, *T. japonicus* is very successful in colonizing this area considering its younger origin in the genus *Trichiurus*, with stable population size following a sudden expansion for more than one magnitude from around 70 to 50 kya. This exponential growth period corresponds to the low temperature at the last ice age, whilst is against the general expectations of expansion with warming after glaciation (Grant, 2015). This lack of concordance with warming was also suggested by mitochondrial cyt b marker (70–216 kya) (He et al., 2014) and implies that the capability to tolerate cold might enable *T. japonicus* to occupy the wide continental shelf in the NW Pacific when the temperature is low. Such a temperature-related niche partitioning could have led to divergence between *T. japonicus* and *T. lepurus* around 14.96 to 13.92 mya, which well corresponds to the timing reported based on cyt b (9.3–13.3) and a temperature-related speciation mechanism previously proposed (Ennyu, 2003; Tzeng, Chen & Chiu, 2007).

## Conclusions

In the two cosmopolitan species, *T. lepturus* and *T. nanhaiensis*, we observed population differentiation corresponding to the coasts of warm oceans which might be contributed by historic geographic barriers in the Pleistocene. In the NW Pacific, the four co-occurring *Trichiurus* species show differences in their fine-scaled distribution range which suggests ecological niche partitioning. The small-sized *T. brevis* is present closer to shore; the warm-affiliated *T. nanhaiensis* is present along the track of the Kuroshio Current; the cold-tolerant *T. japonicus* might have temperature-related niche partitioning and diverged from the widely-distributed *T. lepturus*. Furthermore, a consistent single genetically well-mixing fish stock, thus one management unit, was identified in each of the four species, probably due to the population size expansion predated the Last Glacial Maximum and a lack of distribution barrier. The most dominant *T. japonicus* has at least one magnitude higher effective population size than the others and a unique abrupt size expansion event at 75 to 50-kilo years ago when the low sea level occurred during the ice age. Our genetic analyses not only help delineate the distribution ranges of species and subsequent divisions, but reconstruct the prehistorical events that gave rise to the present population structure. Such information complements research on species distributions such as species distribution modeling (Franklin, 2010) and enables to forecast their ability to resist future environmental and anthropogenic disturbances.

## Supplemental Information

10.7717/peerj.12639/supp-1 Supplemental Information 1 GenBank sequences of the four cutlassfish species and scombroid species included for analyses in this study

10.7717/peerj.12639/supp-2 Supplemental Information 2 Summary of estimated node age (mya) of trichiurids based on two different node constrains The numbers in parentheses are 95% credible interval.

10.7717/peerj.12639/supp-3 Supplemental Information 3 Mismatch distribution observed in samples of the four cutlassfish species and included groups

10.7717/peerj.12639/supp-4 Supplemental Information 4 Maximum likelyhood (ML) tree of trichiurids reconstructed based on 16S Bootstrap values lower than 75 were not shown.

10.7717/peerj.12639/supp-5 Supplemental Information 5 Bayesian inference (BI) tree of trichiurids reconstructed based on 16S Posterior probabilities lower than 85 were not shown.

## Additional Information and Declarations

### Data Availability

The following information was supplied regarding data availability: The 16S ribosomal RNA sequences with GenBank accession numbers MW898456 to MW898458 and MW898460 to MW898578 are uploaded as the supplemental files, EM_flatfile, TL_flatfile, TN_flatfile, TJ_flatfile, and TB_flatfile.
