# Supplementary figures and images for: Variation in global distribution, population structures, and demographic history for four Trichiurus cutlassfishes
Source: PeerJ. 2021 Dec 15;9:e12639. doi: 10.7717/peerj.12639 (PMC8684317; doi:10.7717/peerj.12639)

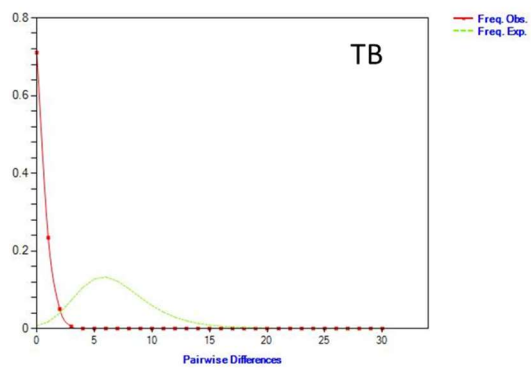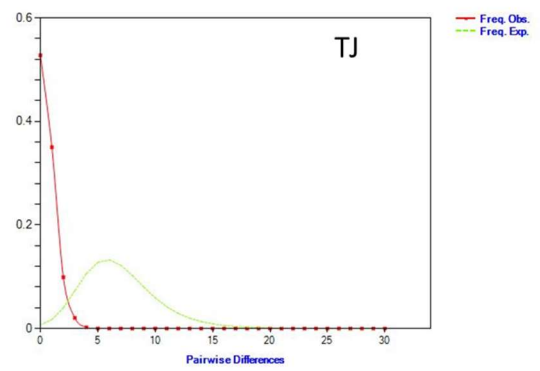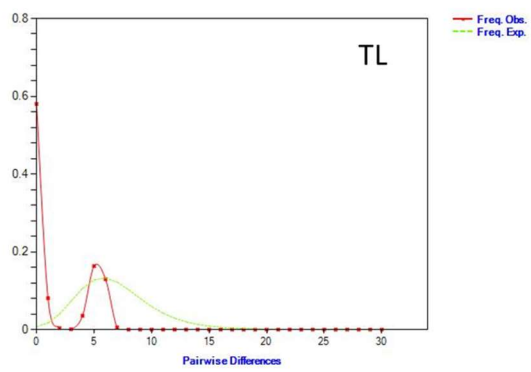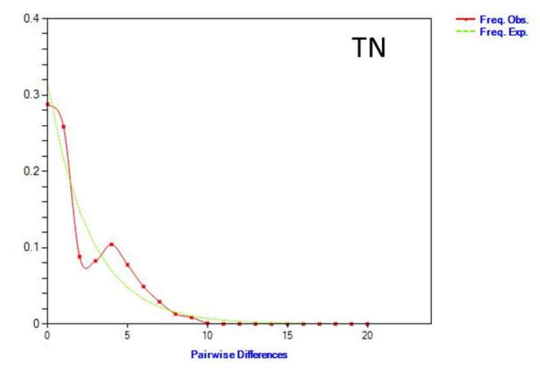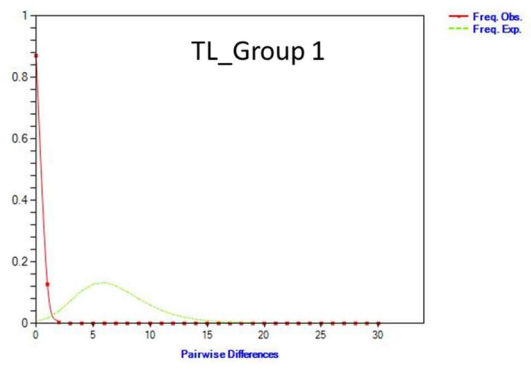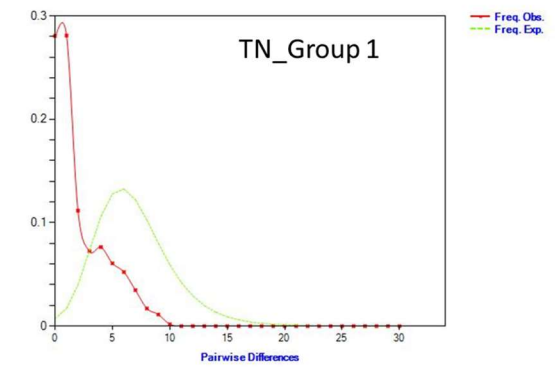

Supplement: Supplemental Information 3 [file peerj-09-12639-s003.pdf]

Tree scale: 0.1

Group

TI-1

TI-2

TI-3

TI-4

Tn-1

Tn-2

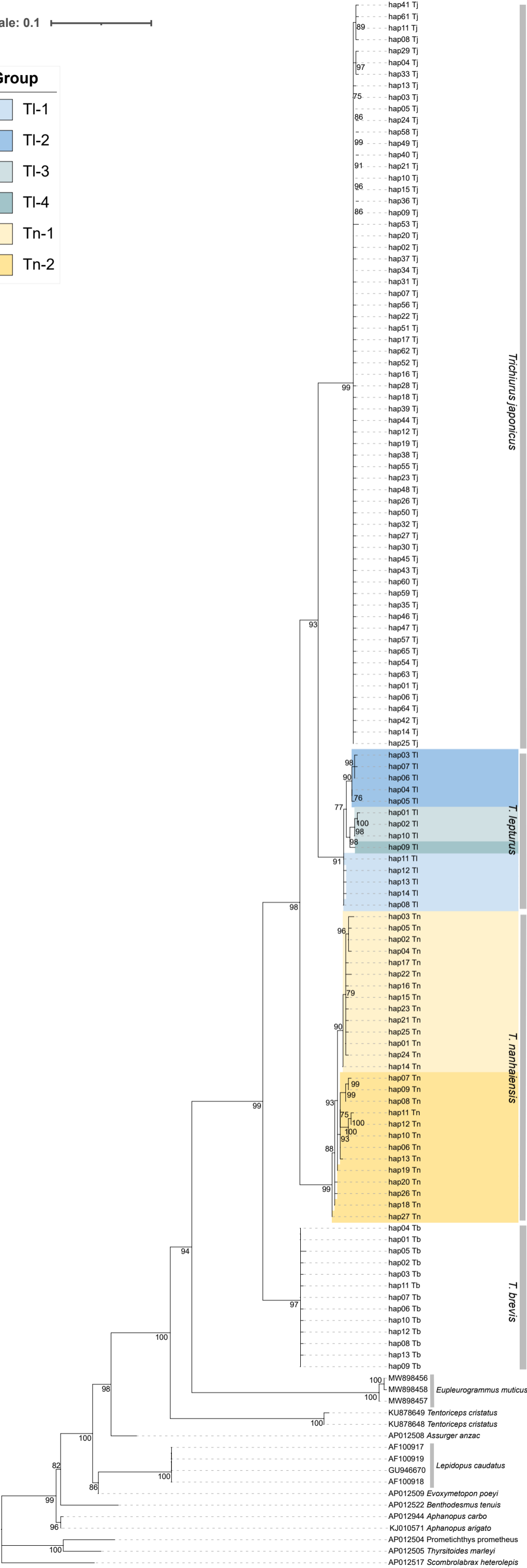

Trichiuridae

outgroup

Supplement: Supplemental Information 4 — Bootstrap values lower than 75 were not shown. [file peerj-09-12639-s004.pdf]
